# *In Planta* Study Localizes an Effector Candidate from *Austropuccinia psidii* Strain MF-1 to the Nucleus and Demonstrates In Vitro Cuticular Wax-Dependent Differential Expression

**DOI:** 10.3390/jof9080848

**Published:** 2023-08-14

**Authors:** Carolina Alessandra de Almeida Hayashibara, Mariana da Silva Lopes, Peri A. Tobias, Isaneli Batista dos Santos, Everthon Fernandes Figueredo, Jessica Aparecida Ferrarezi, João Paulo Rodrigues Marques, Joelma Marcon, Robert F. Park, Paulo José Pereira Lima Teixeira, Maria Carolina Quecine

**Affiliations:** 1 Department of Genetics, “Luiz de Queiroz” College of Agriculture, University of São Paulo, Piracicaba 13418-900, SP, Brazil; carolina.h@usp.br (C.A.d.A.H.); marianasilva_lopes@hotmail.com (M.d.S.L.); izanely@gmail.com (I.B.d.S.); jessica.ferrarezi@usp.br (J.A.F.); joelma.marcon@gmail.com (J.M.); 2 School of Life and Environmental Sciences, The University of Sydney, Camperdown, NSW 2006, Australia; peri.tobias@sydney.edu.au; 3 Center for Nuclear Energy in Agriculture (CENA), University of São Paulo, Piracicaba 134009-70, SP, Brazil; everthon.figueredo@usp.br; 4 Department of Basic Sciences, Faculty of Animal Science and Food Engineering, University of São Paulo, Pirassununga 13635-900, SP, Brazil; jprmarques@usp.br; 5 School of Life and Environmental Sciences, Plant Breeding Institute, The University of Sydney, Cobbitty, NSW 2570, Australia; robert.park@sydney.edu.au; 6 Department of Biological Sciences, “Luiz de Queiroz” College of Agriculture, University of São Paulo, Piracicaba 13418-900, SP, Brazil; paulojt@usp.br

**Keywords:** myrtle rust, eucalypt, cuticular wax, nuclear effectors, *Austropuccinia psidii*

## Abstract

*Austropuccinia psidii* is a biotrophic fungus that causes myrtle rust. First described in Brazil, it has since spread to become a globally important pathogen that infects more than 480 myrtaceous species. One of the most important commercial crops affected by *A. psidii* is eucalypt, a widely grown forestry tree. The *A. psidii–Eucalyptus* spp. interaction is poorly understood, but pathogenesis is likely driven by pathogen-secreted effector molecules. Here, we identified and characterized a total of 255 virulence effector candidates using a genome assembly of *A. psidii* strain MF-1, which was recovered from *Eucalyptus grandis* in Brazil. We show that the expression of seven effector candidate genes is modulated by cell wax from leaves sourced from resistant and susceptible hosts. Two effector candidates with different subcellular localization predictions, and with specific gene expression profiles, were transiently expressed with GFP-fusions in *Nicotiana benthamiana* leaves. Interestingly, we observed the accumulation of an effector candidate, Ap28303, which was upregulated under cell wax from rust susceptible *E. grandis* and described as a peptidase inhibitor I9 domain-containing protein in the nucleus. This was in accordance with in silico analyses. Few studies have characterized nuclear effectors. Our findings open new perspectives on the study of *A. psidii–Eucalyptus* interactions by providing a potential entry point to understand how the pathogen manipulates its hosts in modulating physiology, structure, or function with effector proteins.

## 1. Introduction

*Austropuccinia psidii* is a biotrophic fungus [1] that causes rust disease in species of the Myrtaceae family, known as eucalypt rust, guava rust, ‘ohi‘a rust, or myrtle rust [2]. *A. psidii* is native to South America and was first reported in 1884 on infected native guava (*Psidium guajava* L.) [3], and on introduced eucalypts in 1912 in Brazil [4,5]. Subsequently, myrtle rust has spread around the world [2], including to Australia where it is now a major threat to ecosystems that are home to around 2250 species of endemic Myrtaceae [6]. Despite its specialized biotrophic lifestyle, the pathogen has been reported to infect more than 480 species of Myrtaceae [7,8]. In eucalypt plantations, *A. psidii* causes serious economic losses with damage to young plants and subsequently reduced productivity or resulted in death after successive infections [9,10,11].

Differences in host specialization have been observed in *A. psidii*, implying high genetic diversity among different pathogen populations and strains [12,13]. Although many studies have investigated this question via cross-inoculation experiments [14,15,16,17] and with microsatellite markers [18,19,20], the interaction of various pathogen strains with different hosts remains poorly defined. It is probable that different genotypes of *A. psidii* are host-specific and that they evolved on native South American taxa, which are largely species in the Myrteae tribe [21].

Diversity among global populations of *A. psidii* has been investigated with host-specific and pandemic strains from its native range and with invasive strains across Pacific countries and South Africa [20,21,22]. The assembled genomes of two invasive strains from Australia (pandemic Au-3) and South Africa (PREM) proved to be the largest of all sequenced fungal genomes at 1 Gbp [23,24]. More recently, a phased chromosome-level genome of Au-3 revealed the presence of 18 haploid chromosomes [25] and clarified the tetrapolar mating strategy for *A. psidii* [26]. A third genome assembly was derived from the strain MF-1, which was isolated from *E. grandis* W. Hill ex. Maiden in Brazil. In comparison to Au-3 and PREM, this strain has a smaller predicted genome size (650 Mbp), though this could be due to differences in the sequencing technologies used (GenBank accession number GCA_000469055.1). The MF-1 mitochondrial genome is also available [27] and meiotic spores have been observed in older infections on *E. grandis* [26]. Due to the complexity of *A. psidii* host interactions, in particular the numerous pathogen strains, as well as different host genotypes, much remains to be understood about this important plant pathogen.

Several definitions have been proposed for effector proteins, however, in general they are molecules secreted by an organism that may modify the physiology, structure, and function of another organism [28]. More recently, effectors have been defined as secreted proteins, and other molecules that affect plant physiology in ways that contribute to disease establishment and progression [29]. During plant infection, pathogenic fungi use these molecules to modulate host physiology and colonization [30]. Plants also use their recognition and response mechanisms to counteract the fungal infection, as proposed in the zig-zag model by Jones and Dangl [31]. Santos et al. [32] found evidence from in vitro studies of cuticular wax extract from *Eucalyptus* leaves that the wax was an important pre-formed mechanism against the pathogen. The finding indicated a potential relationship between the cuticular wax composition of resistant and susceptible species and the in vitro germination pattern of *A. psidii*. It is therefore possible that cuticular wax may influence effector expression.

Currently, very little is known about the role of *A. psidii* effectors in pathogenesis on different hosts. In the present study, we generated a comprehensive catalogue of effector candidates from *A. psidii* MF-1, a host-specific strain, and verified the expression of selected gene candidates in vitro in the presence of cuticular waxes from resistant and susceptible *Eucalyptus*. We then transiently expressed a predicted MF-1 effector candidate, Ap28303, in *Nicotiana benthamiana* cells and confirmed its in silico nuclear localization prediction. Interestingly, this effector is characterized as a protease inhibitor I9 domain-containing protein (PF05922), suggesting an inhibitory mechanism for enzymatic cleavage of proteins inside the host nucleus. Our findings provide the first published evidence of subcellular localization for a candidate effector in any myrtle rust study.

## 2. Materials and Methods

### 2.1. Biological Material

*A. psidii* MF-1 strain was initially established from a single rust pustule on *E. grandis* [33]. The MF-1 strain is known to successfully infect different *Eucalyptus* species, as previously evaluated by natural field infection [32]. We used these urediniospores to perform assays of effector candidate expression in vitro using cuticular wax extracted from *E. grandis* and *E. urophylla* leaves. The urediniospores were stored in a –80 °C freezer prior to inoculating leaves of their respective hosts.

*Nicotiana benthamiana* plants for transient expression studies were kept at approximately 28 °C with photoperiod of 16/8 h (light/dark).

*Escherichia coli* and *Agrobacterium tumefaciens* were grown in Luria-Bertani (LB) medium at 37 °C and 28 °C, respectively. For the cloning experiments, *E. coli* DB3.1 and *E. coli* DH5α were used to maintain and propagate plasmids. *A. tumefaciens* GV3101 was used for transient expression assays in *N. benthamiana* leaves. All bacterial strains, including the transformants, were stored in 20% glycerol at −80 °C. Antibiotics to prevent bacterial contamination were used in the following concentrations: kanamycin (50 µg/mL and 100 µg/mL), spectinomycin (50 µg/mL), gentamycin (25 µg/mL and 50 µg/mL), rifampicin (100 µg/mL), and ampicillin (50 µg/mL). All plasmids used in this study are listed in Table 1.

### 2.2. In Silico Prediction of Effector Candidates

**Figure 1:**
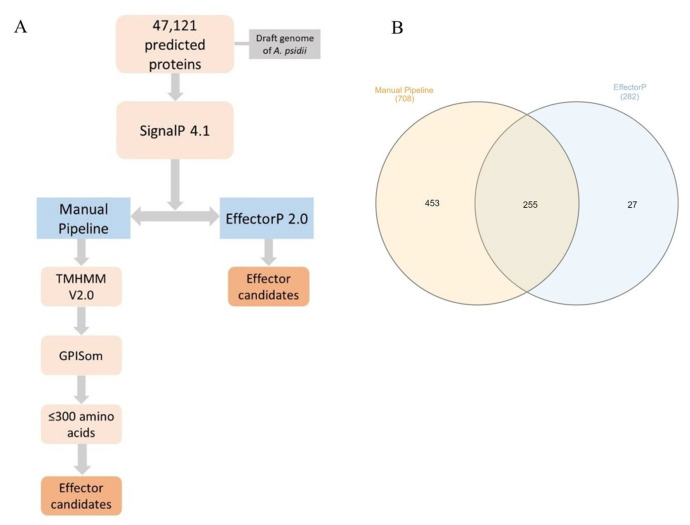
In silico prediction workflow for the prediction of *A. psidii* effector candidates (**A**) and Venn diagram of the effector candidates predicted by manual pipeline (left) and EffectorP (right) and those common to both methods (center) (**B**).

Effector candidates were predicted from an *A. psidii* MF-1 draft genome (NCBI accession number: AVOT00000000). Two methods were used: a manual pipeline and the EffectorP 2.0 software [37] (Figure 1A). First, the 47,121 annotated protein sequences were screened with SignalP 4.1 [38] to predict the presence of a signal peptide. All proteins with predicted signal peptides were then scrutinized using the following two methods: firstly, amino acid sequences were submitted to TMHMM V2.0 [39] and GPISom [40] to check for the transmembrane domain and glycophosphatidylinositol (GPI)-anchors, respectively, using default parameters; secondly, proteins with no transmembrane domain, no GPI-anchor, and with less than 300 residues were selected as described by Duplessis et al. [41] and Germain et al. [42]. We ran protein sequences through the EffectorP 2.0 pipeline using default parameters.

The predicted effectors that were common to both strategies were used for further analysis. The subcellular localization of the predicted effector candidates was determined by LocTree3 [43]. Functional categorization was obtained by the Gene Ontology Consortium (GO) [44], with terms derived from the Blast2GO software using default parameters [45]. The annotation was manually validated using the UniProtKB reference proteomes Swiss-Prot database [46].

### 2.3. Effect of Cuticular Wax on MF-1 Effector Candidate Gene Expression

The modulation of expression of seven effector candidates (Table 2) according to the host was performed using cuticular wax from the leaves of *E*. *grandis* (susceptible) and *E. urophylla* (resistant) [32]. Young leaves were collected and cuticular wax was extracted as described by Viana et al. [47] and adapted by Santos et al. [32]. The treatments comprised Petri dishes with agar water (0.8%) amended with 50 µL of mineral oil, dialysis membrane, 50 µL of cuticular wax extract, and 7 mg of *A. psidii* MF-1 urediniospores. The plates were kept in the dark at room temperature. Sampling intervals were selected based on pathogen in vitro development dynamics: (i) 0 h.p.i—resting urediniospores, (ii) 12 h.p.i—germ-tube formation, and (iii) 24 h.p.i—appressorium and penetration hypha formation [48]. For each sampling interval, we used five biological replicates, each comprising one Petri dish for each species.

### 2.4. RT-qPCR

For cuticular wax stimulus assays, the dialysis membrane with the urediniospores was collected at each interval and ground in liquid nitrogen immediately. RNA was extracted using RNeasy Plant Mini Kit^®^ (Qiagen, Hilden, Germany) following the manufacturer’s protocol. The cDNA synthesis was performed with the RevertAid First Strand cDNA Synthesis Kit^®^ (Thermo Fisher Scientific^TM^, Waltham, MA, USA) following the manufacturer’s protocol.

RT-qPCR was performed with the following parameters: initial denaturation 95 °C for 5 min, 95 °C for 30 s, and 58 °C for 45 s for 35 cycles, and the dissociation curve (95 °C—15 s; 60 °C—30 s; 95 °C—15 s) in the Applied Biosystems 7300 instrument (Applied Biosystems, Waltham, MA, USA). The reaction of 12.5 µL was performed with the GoTaq^®^ qPCR Master Mix (Promega, Madison, WI, USA) composed by GoTaq mix (1×), CXR Reference Dye (300 nM), forward and reverse primers (200 nM each), cDNA sample (2 µl), and nuclease-free water to complete the volume. All treatments included five biological replicates and two technical replicates. We randomly selected hypothetical proteins and genes with known function as effectors to evaluate expression. Two sets of primers were used as reference genes (Supplementary Table S1).

RT-qPCR amplification specificity was checked by dissociation curve analysis (melting curve). The fluorescence-per-cycle data were submitted to LinRegPCR v.11.0 [49] to calculate the average amplification efficiency of each primer set. We used the Relative expression software tool (REST^©^) described by Pfafll et al. [50,51] to analyze our results. This method compares two groups (sample and control). The mathematical model used is based on the PCR efficiencies and the mean crossing point deviation between the sample and control group. The expression ratio results of the investigated transcripts were tested for significance by a randomization test (2000 randomizations). The expression graphics were designed using the R package ggplot [52].

### 2.5. Effector Candidate Gene Cloning

Based on the in vitro gene expression results, five effector candidates were selected for transient expression in *N. benthamiana*. The coding sequences of five effector candidates, here named Ap28303, Ap12491, Ap30385, Ap23389, and Ap15054 (Table 2), were used to manually design primers (Supplementary Figure S1) and the cloning was performed using the Gateway System (Supplementary Figure S2). To construct plasmids for agroinfiltration, effector candidates were PCR amplified using cDNA samples and cloned into the Gateway entry vector, pDONR221 (Invitrogen, CA, USA), and then recombined into the binary destination vectors, pGWB651 (35S::G3GFP-*ccdb*) and pGWB652 (35S::*ccdb*-G3GFP). We generated four constructs: 35S::Ap28303-G3GFP, 35S::Ap30385-G3GFP, 35S::G3GFP-28303, and 35S::G3GFP-Ap30385 from two effector candidates using C- and N-terminal tags. The other three candidates were not successfully cloned. Constructs were confirmed by PCR using M13 primers and sequenced by the Sanger method. All primers used in this work are listed in Supplementary Table S1.

### 2.6. Agroinfiltration-Mediated Transient Expression in N. benthamiana

Expression vectors were electroporated into the *A. tumefaciens* strain, GV3101, according to Win et al. [53]. *Agrobacterium* cells carrying a plasmid with the gene silencing suppressor protein P19 were co-infiltrated at OD_600_ = 0.1, as well as the mcherry plasmids (nucleus and plasmatic membrane markers). *A. tumefaciens* containing the empty plasmids pGWB651 and pGWB652 were used as controls. The infiltrations were performed in *N. benthamiana* leaves using a syringe without a needle.

Transient expression was evaluated two days after infiltration. The agroinfiltrated leaves were collected, cut into sections of approximately 10 mm^2^, and placed in PBS (Phosphate Buffered Saline: 8 g NaCl, 0.2 g KCl, 1.44 g Na_2_HPO_4_, 0.24 g KH_2_PO_4_, and distilled water to complete the volume of 1000 mL, pH 7.4) between a slide and cover glass. The compartments with protein accumulation were verified by laser-scanning microscopy (Nikon Eclipse Ti/C2si, Tokyo, Japan) in the Laboratory of Plant Breeding at CENA/USP.

### 2.7. Protein Extraction and Western Blot Analysis

Two days after agroinfiltration, *N. benthamiana* leaves were harvested and three discs (diameter: 0.9 cm) were extracted and kept at −80 °C until protein extraction. Leaf samples were ground (three times at 30 Hz for 30 s each) in a TissueLyser II bead beater using three 3 mm glass beads. After the addition of 60 µL Protein Extraction Buffer (50 mM Tris-HCl, pH 8.0; 1% SDS; 1 mM EDTA, pH 8.0; Beta-mercaptoethanol; Protease inhibitor; Ultrapure water), the samples were incubated on ice for 5 min. Next, the samples were centrifuged twice at 14,000 rpm for 10 min at 4 °C. Sixty microliters of the supernatant were collected after each centrifugation. Finally, 12 µL of 6× Loading Buffer (375 mM Tris-HCl, pH 6.8; 60% Glycerol; 12% SDS; 0.6 M DTT; 0.06% Bromophenol blue; Ultrapure water) were added to the samples, which were then incubated at 95 °C for 5 min. A total of 20 µL of each sample was separated in a 12% SDS-PAGE gel.

Proteins from the SDS-PAGE gel were transferred to a nitrocellulose membrane using a semi-dry system (Owl^TM^, Thermo Scientific^TM^, Waltham, MA, USA). Membrane blocking was performed using 5% skim milk powder in 1× TBS-T buffer (10× TBS; Ultrapure water; Tween 20) for 1 h at room temperature, after which the membrane was washed three times in TBS-T for 5 min. The membrane was then incubated with a rabbit anti-GFP antibody (GFP Polyclonal Antibody, Invitrogen by Thermo Scientific^TM^, Waltham, MA, USA) at a dilution of 1:1000 in 10 mL of 1% skim milk powder overnight at 4 °C. The membrane was then washed three times with TBS-T and incubated for 1 h at room temperature with a 1:5000 dilution of a Goat anti-Rabbit secondary antibody (IgG (H+L) Cross-Adsorbed Secondary Antibody, HRP, Invitrogen by Thermo Scientific^TM^, Waltham, MA, USA). Then, the membrane was washed three times in TBS-T and once in 1× TBS to remove residual Tween 20. The signal was visualized with the Amersham ECL Prime reagent (GE Healthcare, Chicago, IL, USA) according to the manufacturer’s instructions and revealed in the ChemiDoc XRS+ Gel Imaging System (Bio-Rad, Hercules, CA, USA).

## 3. Results

### 3.1. In Silico Analyses Identify 255 Effector Candidates in the Genome of A. psidii Strain MF-1

We identified 708 and 282 effector candidates using our manual pipeline and EffectorP 2.0, respectively. From these, 255 effector candidates were common to both approaches (Figure 1B) and were used in subsequent studies (Supplementary Table S2). Based on LocTree3 predictions, the most abundant subcellular localization of effector candidates was apoplastic (87.45%), followed by cytoplasmic (6.27%) and nuclear (3.14%) (Table 3).

**Figure 2:**
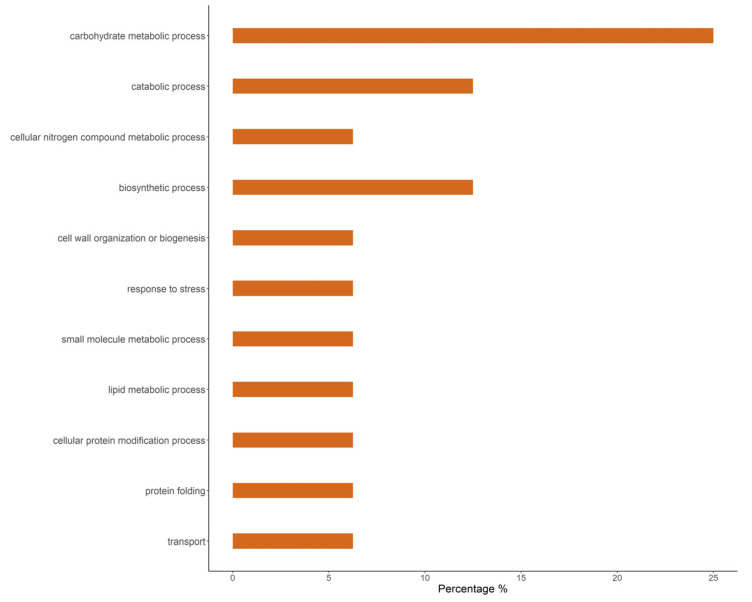
Gene ontology for biological process GO terms obtained with Blast2GO (Conesa et al., 2005) of *A. psidii* effector candidates. The bar graph represents the percentage composition of terms in the effector candidates.

Most (65.49%) effector candidates had no annotation assigned or were generically annotated as hypothetical proteins (23.14%). Only 11.37% were annotated with a known function. Among the effector candidates with known function, we identified enzymes including hydrolases, lipases, and chitin deacetylases (Supplementary Table S2). A majority of the effector candidates of known function shared those functions with other rust species (*Puccinia* spp. and *Melampsora larici-populina*) (Table 4). Finally, according to Blast2GO, the most abundant biological process terms were carbohydrate metabolic processes, catabolic processes, and biosynthetic processes (Figure 2).

### 3.2. Effect of Cuticular Wax on MF-1 Effector Candidate Gene Expression

**Figure 3:**
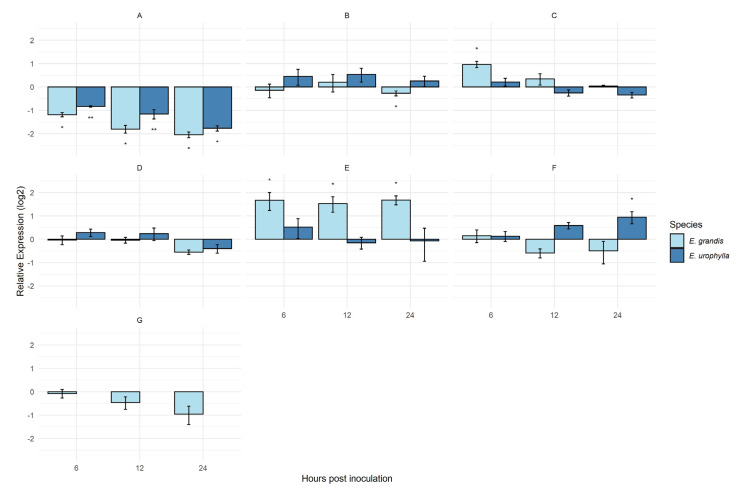
MF-1 strain expression of effector candidates under cuticular wax extracts from *E. grandis* and *E. urophylla* at 6, 12, and 24 h post-inoculation; The time 0 h post-inoculation was considered as control and the transcript levels of effector candidates were normalized to the levels of the reference gene beta-tubulin (*Btub*) and effector elongation (*EF*) to perform the analyses in REST; effector candidate Ap15054 (**A**); effector candidate Ap30385 (**B**); effector candidate Ap28303 (**C**); effector candidate Ap11108 (**D**); effector candidate Ap12491 (**E**); effector candidate Ap23389 (**F**); effector candidate Ap2160 (**G**). * *p* < 0.05; ** *p* < 0.001.

From the 255 effector candidates, seven were selected to evaluate their expression (Table 2). The expression of Ap15054 was significantly downregulated at all time points for both cuticular wax sources (Figure 3A). At 6 h.p.i., the genes, Ap28303 and Ap12491, were upregulated and showed a differential expression when urediniospores were germinated in the presence of the wax of the susceptible host *E. grandis* (Figure 3C,E). The effector candidates, Ap30385 and Ap28303 (Figure 3B,C), were differentially expressed at one timepoint only under the cuticular wax stimulus of *E. grandis.* Effector candidate Ap1108 (Figure 3D) did not show significant expression under cuticular wax stimulus at any timepoint sampled. The only effector candidate differentially expressed and upregulated at all timepoints was Ap12491, under the stimulus of cuticular wax from *E. grandis* (Figure 3E). Ap23389 under the cuticular wax stimulus of *E. urophylla* was differentially expressed at 24 h.p.i (Figure 3F). The expression of the effector candidate Ap2160 was not detected under the stimulus of *E*. *urophylla* cuticular wax (Figure 3G), only under the stimulus of *E. grandis* cuticular wax.

### 3.3. Subcellular Localization of Effector Candidate Ap28303

**Figure 4:**
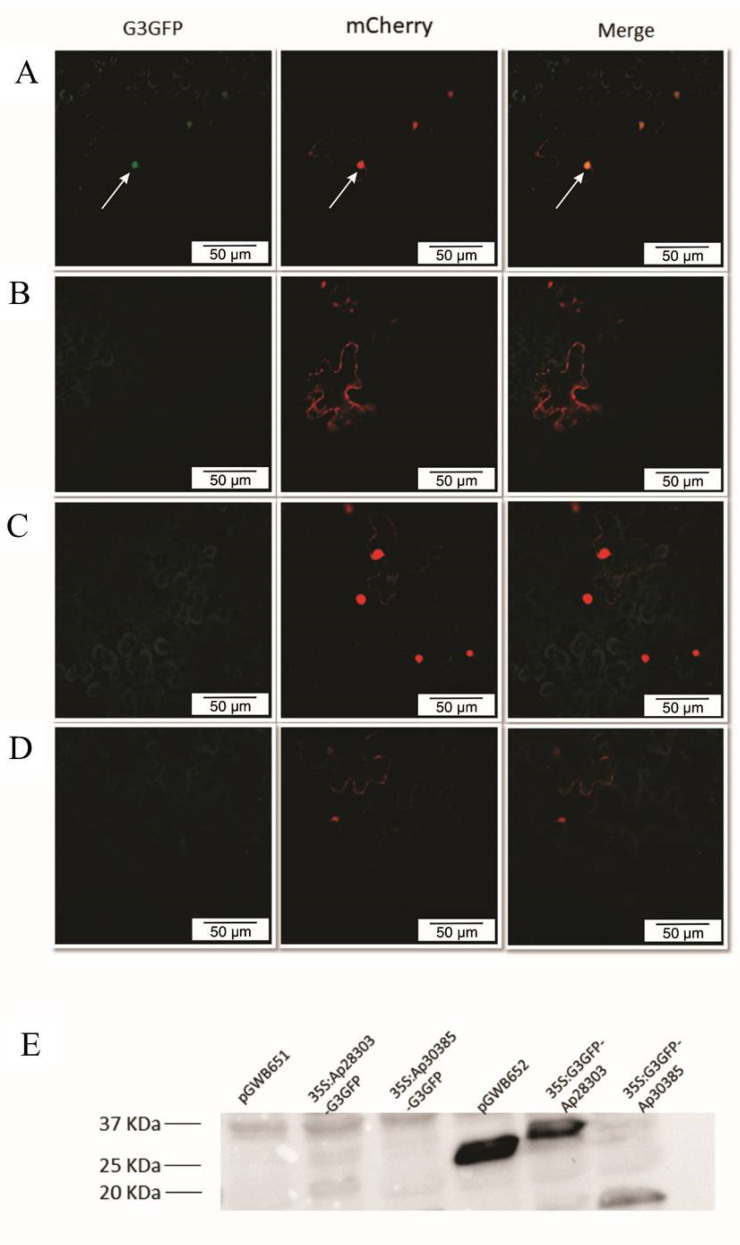
Confocal microscopy of the effector candidates (**A**) 35S::G3GFP-Ap28303 construct accumulated in the nucleus. White arrows indicate nuclei; (**B**) no accumulation of 35S::Ap28303-G3GFP; (**C**) no accumulation of 35S::G3GFP-Ap30385; (**D**) no accumulation of 35S::Ap30385-G3GFP; and (**E**) a band of the expected size for the 35S::G3GFP-Ap28303 fusion (40 KDa: 13 kDa of Ap28303 protein + 27 kDa of GFP protein). As expected, expression of the empty pGWB652 vector resulted in a band compatible with the G3GFP tag (~27 kDa).

We observed nuclear accumulation of the Ap28303 effector candidate in *N. benthamiana* leaves transiently expressing the 35S::G3GFP-Ap28303 construct (Figure 4A). No fluorescence was detected when a C-terminal GFP tag was used (35S::Ap28303-G3GFP) (Figure 4B). On the other hand, accumulation of Ap30385 was not observed by confocal microscopy regardless of the fusion tag used (35S::Ap30385-G3GFP and 35S::G3GFP-Ap30385) (Figure 4C,D). In agreement with these results, protein accumulation could only be detected by Western Blot in plants expressing the GFP-Ap28303 construct (Figure 4E).

All attempts to clone the Ap12491, Ap23389, and Ap15054 effector candidates were unsuccessful. We successfully cloned the effector candidate, Ap30385, with the *A. tumefaciens* binary plasmid but did not observe accumulation in any subcellular compartment.

## 4. Discussion

### 4.1. Two Methods of In Silico Effector Characterization for Austropuccinia psidii Strain MF-1 Commonly Predicted 255 Candidate Genes

We identified 255 candidate effectors in the *A. psidii* MF-1 strain haploid genome based on manual curation and the Effector P 2.0 pipeline. Candidate effector predictions by Effector P 2.0 in the pandemic *A. psidii* isolate (Au-3) identified an average of 335 effectors [24], while the more recent Au-3 genome indicated 616 effectors [25].

It is known from studies of other rust pathogens that the number of effectors predicted varies according to the criteria and/or method used, as well as being based on strain. In *P. striiformis* f. sp. *tritici* (the wheat stripe rust pathogen), 969 effector candidates were predicted for the Pst-104E strain by EffectorP 1.0 [54], and 557 effector candidates were predicted for the Pst-DK0911 strain using EffectorP 2.0 [55]. Garnica et al. [56] found 437 haustorial secreted proteins using a manual pipeline, whereas our results showed a higher number of effector candidates predicted by the manual pipeline compared to the software. Clearly, the number of effector candidates predicted in rust pathogen genomes is variable and may be related to genome size, the type of sequence data used in an assembly, the host–pathogen interaction, and/or the prediction method used. Thus, the difference in the number of effectors predicted in our work may be a result of the genome sequence data and the search methods. We were nonetheless very conservative in our approach, selecting only effector candidates identified by both methods via posteriori analysis.

Our results showed that *A. psidii* MF-1 carried a higher number of predicted apoplastic effectors compared to other rusts. We predicted 87% apoplastic effectors in MF-1, compared to results from Sperschneider et al. [57] in *P. striiformis* f. sp. *tritici* PST-130 (61.2%), *P. graminis* f. sp. *tritici* (61.6%), *M. larici-populina* (58.7%), and *P. triticina* (53.51%). The average amount of secreted proteins from rust fungi for targeting different organelles were approximately 3.35% chloroplast, 0.93% mitochondria, and 4.5% nucleus. The proportion of effector candidates targeting the nucleus was the highest in *P. graminis* f. sp. *tritici* (5.61%) and *P. triticina* (5.44%). We found 3.14% of effector candidates targeting the nucleus. A few studies have shown translocation of effector proteins secreted by fungal pathogens into the plant nucleus [58,59].

In predicting the target location of effectors, Caillaud et al. [60] found that only 20% of those predicted in silico and in vivo had the same location. Therefore, prediction in silico is an essential step to confirm the effector candidates’ localization.

### 4.2. Host Leaf Cuticular Wax Changes In Vitro Pathogen Effector Gene Expression

Cuticular waxes contribute to plant defense and resistance against pathogenic fungi, and, consequently, are effector targets. The effector, *cutinase2,* from *Magnaporthe grisea* is activated during host penetration [61] (Skamnioti and Gurr, 2007). Santos et al. [32] used cuticular waxes from eucalypt species as stimuli in deciphering pathogenesis-related mechanisms during infection of different host species by *A. psidii*. We therefore investigated host modulation of effector expression by an expression analysis of the MF-1 strain under the stimulus of cuticular wax from a resistant and a susceptible eucalypt species.

We found that cuticular wax can modulate the expression of candidate effector MF-1. A previous study has shown the importance of host cuticular wax during infection for recognition, penetration, and plant resistance [62], however, our results appear to be the first to establish an interaction between effector expression and host cuticular wax in suggesting that cuticular wax acts as a stimulus for the development of *A. psidii*.

Interestingly, the effector candidate, Ap11108, did not show differential expression when *A. psidii* strain MF-1 was exposed to cuticular wax. Ap11108 was shown to be a homolog of a chorismate mutase domain-containing protein of *P. coronata* var. *avenae* f. sp. *avenae* (Supplementary Table S2). The effector, *Cmu1*, was described in *Ustilago maydis* as a secreted chorismate mutase by Djamei et al. [63], who suggested that it played a role in virulence by impacting the conversion of plant cell chorismate to suppress the salicylic acid immune pathway. This mechanism is important for allowing biotrophic fungi to avoid plant defenses [64].

Ap23389 was upregulated only at 24 h.p.i under the stimulus of cuticular wax from the resistant species, *E. urophylla*. This indicates that this effector, a hypothetical protein localized to the nucleus, might have a role in later stages of infection in resistant genotypes and should be investigated further.

Effector candidate Ap15054 was downregulated and differentially expressed at all time points by cuticular wax from both eucalypt species. According to Oliveira-Garcia and Deising [65], the downregulation of an effector gene may be tied to avoiding recognition by the host. The downregulation of Ap15054 does not exclude it from being an effector. It is known that the upregulation of an effector candidate in planta may not be evidence of whether a protein is an effector or not. Even effectors predicted to be expressed in haustoria were found to be downregulated, suggesting that the post-transcriptional regulation may control effector expression [66].

The effector candidate, Ap12491, a predicted secreted hypothetical protein, was significantly upregulated in the presence of cuticular wax of *E. grandis.* It was the only effector significantly expressed at all intervals under the susceptible cuticular wax stimulus. Similarly, Swanepoel et al. [67] observed seven uniquely expressed candidate effectors over the course of infection within susceptible *E. grandis*. We were unable to proceed with the transient expression of this gene, however, future studies should investigate this effector candidate in greater detail.

The effector candidate, Ap28303, was identified as an inhibitor I9 domain-containing protein. The in vitro assay showed that Ap28303 was upregulated and differentially expressed at 6 h.p.i. under the cuticular wax stimulus of the susceptible species, *E. grandis.* Thus, the expression of Ap28303 might be stimulated during the early stages, establishing a compatible infection in a susceptible host. Interestingly, Ap28303 was predicted to be a nuclear-localized effector based on in silico subcellular localization. Most effectors with protease inhibition activity have been localized to the cytoplasm of infected hosts [66]. However, the knowledge of the molecular mechanism of the nuclear effector of phytopathogenic fungi in plant disease is limited [68].

### 4.3. In Planta MF-1 Effector Candidate Localized to the Nucleus

Discovering the host subcellular localization of effectors is fundamental to understanding their function [69]. Thus, we first predicted the subcellular localization in silico of effector candidates from *A. psidii* MF-1. Two of these were then expressed transiently, of which the protease inhibitor I9 family member, Ap28303, was successfully visualized as targeting the nucleus.

Plants use proteases in the apoplast to defend themselves, and pathogen effectors are known to target these proteins in order to facilitate successful infection [70]. Several studies have found pathogen effectors that attack proteases from plants [71,72]. Rawlings et al. [73] classified peptidases into 48 families based on similarity, with the family I9 classified as protease inhibitors. In the rust fungi, *U. fabae* and *U. striatus*, the protein RTP1p was found in the cytoplasm and exhibited protease inhibitor activity. The homologues of RTP1p represent a rust fungi-specific family of effector proteins with protease inhibitory activity, which might be associated with effector function during biotrophic interactions [74].

The prediction by LocTree3 showed nuclear localization of Ap28303, which is inconsistent with the classical function of protease inhibitors as established in previous studies. We expressed this fluorescently tagged effector candidate transiently using a binary system in *N. benthamiana* leaves. Using confocal microscopy, we observed its accumulation in the epidermal cell nucleus, confirming our prediction *in silico*.

Effectors targeting the host nucleus have been previously reported [75,76], including in the rust pathogens *M. larici-populina* [77], *Uromyces fabae* [66], and others [68]. Proteins have been known to localize in plant nuclei target transcription factors [78], RNAi components [79], or associate with TOPLESS-related proteins, which are involved in plant immune responses [77]. Moreover, few fungal effectors have been shown to target the plant nuclear functions without being localized into the nucleus [68]. These effector proteins interact with plant transcription factors and alter their nuclear localization, leading to disruption of defense responses. For example, *P. striiformis* f. sp. *tritici* effector Pst GSRE1 was shown to interact with the ROS promoting transcription factor, TaLOL2, disrupting its nuclear localization in plant cells and thereby resulting in loss of the host defense response and increasing fungal proliferation [66].

Future analyses should be directed at understanding the functionality of Ap28303 on the host nucleus. Investigation of this candidate effector’s interaction with host targets, and the biochemical nature of nuclear effects in *Eucalyptus* cells, may be important for the development of novel strategies to prevent disease.

## 5. Conclusions

This pioneering study of effector candidates from *A. psidii* identified 255 effector candidates in the MF-1 genome. In view of the technical difficulties and complexity of the *A. psidii–Eucalyptus* pathosystem, our in vitro assays allowed us to simulate conditions as close as possible to those in planta in order to provide insights into molecular mechanisms during host infection. Our results revealed that cuticular waxes from eucalypt species, resistant and susceptible to *A. psidii*, altered the expression of effector candidates.

One effector candidate, Ap28303, showed nuclear localization in a heterologous plant system. This interesting finding warrants further study to fully define the role of Ap28303 during the infection of eucalypts. Although it is annotated as a protease inhibitor, we observed it to be targeted to the host nucleus. This finding might imply more than one function or a different function and localization for this effector candidate; for example, interaction with a host transcription factor, as found for Pst GSRE1. This is the first study to report the subcellular localization of effectors in *A. psidii* and is a step forward in the study of genes associated with infection in myrtle rust disease.

## Figures and Tables

**Table 1:** Description of the plasmids used in this work.

Plasmid	Description	Reference
pDONR221	Gateway donor plasmid	Invitrogen, CA, USA
pGWB651	Binary plasmid for C-terminal G3GFP (green fluorescence protein) fusions	[34]
pGWB652	Binary plasmid for N-terminal G3GFP (green fluorescence protein) fusions	[34]
p19	Binary plasmid containing the p19 protein from tomato bushy stunt virus, silencing suppressor of gene expression in tobacco	Provided by the Laboratory of Genetics and Immunology of Plants—ESALQ/USP
pUFV2224	Nuclear marker *Arabidopsis thaliana* AtWWP1 fused to mCherry	[35]
pCMU-PMr; pCMB-PMr	Plasma membrane marker, PIP2a-mcherry; AtPIP2a, plasma membrane aquaporin	[36]
pENTRY::Ap28303	PCR purified product from the effector candidate Ap28303 recombined in pDNOR221 plasmid	This study
pENTRY::Ap30385	PCR purified product from the effector candidate Ap30385 recombined in pDNOR221 plasmid	This study
35S::Ap30385-G3GFP	pGWB651, binary plasmid for C-terminal G3GFP (green fluorescence protein) fused to the effector candidate Ap30385	This study
35S::G3GFP-Ap30385	pGWB652, binary plasmid for N-terminal G3GFP (green fluorescence protein) fused to the effector candidate Ap30385	This study
35S::Ap28303-G3GFP	pGWB651, binary plasmid for C-terminal G3GFP (green fluorescence protein) fused to the effector candidate Ap28303	This study
35S::G3GFP-Ap28303	pGWB652, binary plasmid for N-terminal G3GFP (green fluorescence protein) fused to the effector candidate Ap28303	This study

**Table 2:** Predicted functions and localizations of predicted effector candidates selected for validation and the number of gene accession.

Effector Candidate Abbreviation	Description ^a^	Localization ^b^	GenBank Accession Number
Ap15054	Secreted protein	Secreted	AVOT02014902.1
Ap30385	hypothetical protein	Nucleus	AVOT02029768.1
Ap28303	inhibitor I9 domain-containing protein	Nucleus	AVOT02027753.1
Ap11108	chorismate mutase domain-containing protein	Cytoplasm	AVOT02011022.1
Ap12491	hypothetical protein	Secreted	AVOT02012383.1
Ap23389	hypothetical protein	Nucleus	AVOT02023014.1
Ap2160	non-annotated	Secreted	AVOT02002154.1

^a^ The description was obtained by Blast2GO and validated by UniProt; ^b^ The localizations were obtained by LocTree3.

**Table 3:** Effector candidate localization in the host predicted by LocTree3 [43].

Localization	Percentage of Hits (%)
Apoplast/secreted	87.45
Cytoplasm	6.27
Nucleus	3.14
Endoplasmatic reticulum	0.78
Mitochondria	0.78
Vacuole	0.78
Chloroplast	0.39
Plasma membrane	0.39

**Table 4:** Description of effector candidates with known function.

Access Number GenBank	Description ^a^
AVOT02009987.1	Alpha, alpha-trehalose-phosphate synthase (UDP-forming) *P. coronata* var. *avenae* f. sp. *Avenae*
AVOT02011022.1	Chorismate mutase domain-containing protein *P. coronata* var. *avenae* f. sp. *Avenae*
AVOT02011687.1	SCP domain-containing protein *P. coronata* var. *avenae* f. sp. *Avenae*
AVOT02011695.1	Secreted protein *M. larici-populina*
AVOT02012399.1	Protein ROT1 *M. larici-populina*
AVOT02066599.1	Tnp4 domain-containing protein *P. striiformis*
AVOT02013624.1	SCP domain-containing protein *P. coronata* var. *avenae* f. sp. *Avenae*
AVOT02013985.1	Secreted protein *M. larici-populina*
AVOT02014902.1	Secreted protein *M. larici-populina*
AVOT02136828.1	Secreted protein *M. larici-populina*
AVOT02015524.1	Chitin deacetylase *P. graminis* f. sp. *tritici*
AVOT02016858.1	SCP domain-containing protein *P. striiformis*
AVOT02017392.1	Carboxylic ester hydrolase *Helicocarpus griseus*
AVOT02002958.1	Chitin deacetylase *M. larici-populina*
AVOT02002632.1	Sod_Cu domain-containing protein *P. graminis* f. sp. *tritici*
AVOT02026132.1	Chitin deacetylase *P. graminis* f. sp. *tritici*
AVOT02027753.1	Inhibitor I9 domain-containing protein
AVOT02028049.1	Secreted protein *M. larici-populina*
AVOT02028690.1	Lipase_3 domain-containing protein *P. striiformis* f. sp. *tritici*
AVOT02029865.1	Sod_Cu domain-containing protein *P. graminis* f. sp. *tritici*
AVOT02030696.1	Dimer_Tnp_hAT domain-containing protein
AVOT02032858.1	Alpha-galactosidase *P. graminis* f. sp. *tritici*
AVOT02035871.1	Alpha/Beta hydrolase protein *Pseudomassariella vexata*
AVOT02037137.1	Thioredoxin domain-containing protein *P. triticina*
AVOT02041469.1	Secreted protein *M. larici-populina*
AVOT02045600.1	DPBB_1 domain-containing protein *P. striiformis* f. sp. *tritici* PST-78
AVOT02048724.1	Secreted protein *M. larici-populina*
AVOT02053223.1	SurE domain-containing protein *P. graminis* f. sp. *tritici*
AVOT02000055.1	Phosphatidylglycerol/phosphatidylinositol transfer protein *P. graminis* f. sp. *Tritici*

^a^ The description was obtained by Blast2GO and validated manually by UniProt.

## Supplementary Materials

The following supporting information can be downloaded at: https://www.mdpi.com/article/10.3390/jof9080848/s1. Table S1: List of primers used in expression validation and cloning experiments (Ref. [80] is cited in Table S1); Table S2: List of 255 effector candidates, subcellular localization predicted by LocTree3 and their functional description by Blast2GO and validated in UniProt; Figure S1: Construction of primers for cloning experiments (a) construction of the primers to be recombined in the plasmid with the tag in C-terminal (b) construction of the primers to be recombined in the plasmid with the tag in N-terminal; Figure S2: Cloning procedures using Gateway system.
