# Supplementary material for: In Planta Study Localizes an Effector Candidate from Austropuccinia psidii Strain MF-1 to the Nucleus and Demonstrates In Vitro Cuticular Wax-Dependent Differential Expression
Source: J Fungi (Basel). 2023 Aug 14;9(8):848. doi: 10.3390/jof9080848 (PMC10455828; doi:10.3390/jof9080848)
Supplement: Supplementary file 1 [file jof-09-00848-s001.zip › jof-2490638-supplementary.pdf]

Supplementary Table S1. List of primers used in expression validation and cloning experiments

Effector candidate/genes	Primer Forward (5'-3')	Primer Reverse (5'-3')	Reference
	Expression Validation		
Ap28303	CCTCAGTTGAACTCGGTGATTC	ATTTGGGGGTTCTGTTGGAG	This study
Ap11108	AAGCATCCAAGCAAGAGGTC	TTCTTGGCAACGTCTTCGTC	This study
Ap15054	GACAGCGAGGAAAATTGCAC	TACGCCCGAAGTTTTACCTG	This study
Ap2160	CTCGAGTGTCCAGGTCCAT	TTAGCGCCTACTCTGGTGCT	This study
Ap12491	TAGCGCCTACTCTGGTGGTT	GCGGATTTTGACGTTT	This study
Ap23389	CGTCGATAGAGCTGCAAACA	GTCCTGGACTAGGTGGTGGA	This study
Ap30385	TCATCCACCATCCTTCAACG	ACTCTCTCACATGCTTCCCAAG	This study
Btub	GGACTCTGTTTTAGATGTCGTC	TTGATGGACTGATAGGGTAGCG	[80]
EF	CAGTTATGGAAGTTTGAAACTCC	GACAATAAGCTGTCGAACACCAAGG	[80]
	Cloning experiments		
Ap30385 (C-terminal)	AGCAGGCTTCACCATGATCGATCCAACGATTTTAATCG	GAAAGCTGGGTCGATAAACTCAAAATAGTCCTTTC	This study
Ap28303 (C-terminal)	AGCAGGCTTCACCATGCAGTTTCAATCTTCTCCTTC	GAAAGCTGGGTCCGAGGTATGAACTTCTCCAT	This study
Ap30385 (N-terminal)	AGCAGGCTTCACCATCGATCCAACGATTTTAATCG	GAAAGCTGGGTCTCAGATAAACTCAAAATAGTCCTTTC	This study
Ap28303 (N-terminal)	AGCAGGCTTCCAGTTTCAATCTTCTCCTTC	GAAAGCTGGGTCTCACGAGGTATGAACTTCTCCAT	This study
Attb	GGGGACAAGTTTGTACAAAAAAGCAGGCT	GGGGACCACTTTGTACAAGAAAGCTGGGT	This study
M13	GTAAAACGACGGCCAG	CAGGAAACAGCTATGAC	This study

attb site; Kozak sequence; Artificial methionine; Stop codon

Supplementary Table S2. List of 255 effector candidates, subcellular localization predicted by LocTree3 and their functional description by Blast2GO and validated in UniProt

NCBI number access	Localization class	Description
AVOT02009987.1	cytoplasm	Alpha,alpha-trehalose-phosphate synthase (UDP-forming) *P. coronata var. avenae* f. sp. *avenae*
AVOT02010077.1	secreted	Hypothetical protein *P. graminis* f. sp. *tritici*
AVOT02010149.1	secreted	---NA---
AVOT02010270.1	secreted	Hypothetical protein *P. striiformis* f. sp. *tritici* PST-78
AVOT02010322.1	secreted	---NA---
AVOT02097545.1	secreted	---NA---
AVOT02010385.1	secreted	Hypothetical protein *P. sorghi*
AVOT02000105.1	secreted	---NA---
AVOT02010452.1	secreted	Hypothetical protein *P. graminis* f. sp. *tritici*
AVOT02010489.1	secreted	---NA---
AVOT02001059.1	secreted	---NA---
AVOT02001073.1	secreted	---NA---
AVOT02011022.1	cytoplasm	Chorismate mutase domain-containing protein *P. coronata* var. *avenae* f. sp. *avenae*
AVOT02011026.1	secreted	Hypothetical protein *P. graminis* f. sp. *tritici*
AVOT02011088.1	secreted	---NA---
AVOT02011120.1	secreted	Hypothetical protein *P. graminis* f. sp. *tritici*
AVOT02011140.1	secreted	Hypothetical protein *P. graminis* f. sp. *tritici*
AVOT02011181.1	secreted	Hypothetical protein *P. graminis* f. sp. *tritici*

AVOT02000011.1	secreted	Hypothetical protein *M. larici-populina*
AVOT02011333.1	secreted	---NA---
AVOT02106808.1	secreted	---NA---
AVOT02011589.1	secreted	Hypothetical protein *P. sorghi*
AVOT02001171.1	secreted	SCP domain-containing protein *P. coronata* var. *avenae* f. sp. *avenae*
AVOT02011687.1	secreted	Secreted protein *M. larici-populina*
AVOT02011695.1	secreted	---NA---
AVOT02011813.1	secreted	---NA---
AVOT02011984.1	secreted	---NA---
AVOT02012065.1	secreted	---NA---
AVOT02012087.1	secreted	---NA---
AVOT02012097.1	secreted	---NA---
AVOT02114164.1	secreted	---NA---
AVOT02012383.1	secreted	Hypothetical protein *P. sorghi*
AVOT02012399.1	secreted	Protein ROT1 *M. larici-populina*
AVOT02012502.1	secreted	Hypothetical protein *P. graminis* f. sp. *tritici*
AVOT02117569.1	secreted	---NA---
AVOT02012631.1	secreted	---NA---
AVOT02066599.1	secreted	Tnp4 domain-containing protein *P. striiformis*
AVOT02012683.1	secreted	---NA---
AVOT02118281.1	secreted	---NA---
AVOT02159210.1	secreted	---NA---
AVOT02122542.1	secreted	---NA---
AVOT02013294.1	secreted	---NA---
AVOT02013324.1	secreted	---NA---
AVOT02124464.1	secreted	---NA---
AVOT02124464.1	secreted	---NA---
AVOT02013507.1	secreted	---NA---
AVOT02013521.1	secreted	Hypothetical protein *P. graminis* f. sp. *tritici*

AVOT02013624.1	secreted	SCP domain-containing protein *P. coronata* var. *avenae* f. sp. *avenae*
AVOT02013627.1	secreted	Hypothetical protein *P. striiformis*
AVOT02000139.1	secreted	---NA---
AVOT02013805.1	secreted	Hypothetical protein *P. coronata* var. *avenae* f. sp. *avenae*
AVOT02001399.1	secreted	---NA---
AVOT02013985.1	secreted	Secreted protein *M. larici-populina*
AVOT02014337.1	secreted	---NA---
AVOT02014415.1	secreted	---NA---
AVOT02014818.1	secreted	---NA---
AVOT02014850.1	secreted	---NA---
AVOT02014902.1	secreted	Secreted protein *M. larici-populina*
AVOT02136828.1	cytoplasm	Secreted protein *M. larici-populina*
AVOT02015021.1	nucleus	---NA---
AVOT02001518.1	cytoplasm	---NA---
AVOT02015185.1	secreted	---NA---
AVOT02015219.1	secreted	---NA---
AVOT02001540.1	secreted	---NA---
AVOT02139845.1	secreted	---NA---
AVOT02015313.1	secreted	Hypothetical protein *P. triticina*
AVOT02015356.1	secreted	---NA---
AVOT02015463.1	secreted	---NA---
AVOT02015524.1	cytoplasm	Chitin deacetylase *P. graminis* f. sp. *tritici*
AVOT02015636.1	secreted	Hypothetical protein *P. coronata* var. *avenae* f. sp. *avenae*
AVOT02015774.1	secreted	---NA---
AVOT02001597.1	secreted	---NA---
AVOT02145259.1	nucleus	Hypothetical protein *P. sorghi*
AVOT02016244.1	secreted	---NA---
AVOT02016245.1	secreted	---NA---
AVOT02012065.1	secreted	---NA---

AVOT02016490.1	secreted	---NA---
AVOT02150253.1	secreted	---NA---
AVOT02019745.1	secreted	---NA---
AVOT02019202.1	vacuole	SCP domain-containing protein *P. striiformis*
AVOT02018607.1	secreted	Hypothetical protein *P. striiformis* f. sp. *tritici*
AVOT02018564.1	secreted	---NA---
AVOT02018433.1	secreted	---NA---
AVOT02017706.1	secreted	---NA---
AVOT02017598.1	secreted	---NA---
AVOT02017555.1	secreted	---NA---
AVOT02002958.1	secreted	---NA---
AVOT02001764.1	secreted	Carboxylic ester hydrolase *Helicocarpus griseus*
AVOT02017392.1	secreted	---NA---
AVOT02001743.1	cytoplasm	Chitin deacetylase *M. larici-populina*
AVOT02017268.1	secreted	---NA---
AVOT02017218.1	secreted	Hypothetical protein *P. striiformis*
AVOT02017179.1	secreted	---NA---
AVOT02017148.1	vacuole	---NA---
AVOT02001713.1	secreted	---NA---
AVOT02016881.1	secreted	Hypothetical protein *P. graminis* f. sp. *tritici*
AVOT02016858.1	secreted	---NA---
AVOT02016596.1	secreted	---NA---
AVOT02019749.1	secreted	---NA---
AVOT02019794.1	secreted	---NA---
AVOT02019969.1	secreted	Hypothetical protein *P. coronata* var. *avenae* f. sp. *avenae*
AVOT02020152.1	secreted	---NA---
AVOT02011984.1	secreted	---NA---
AVOT02020200.1	secreted	---NA---
AVOT02020294.1	secreted	---NA---

AVOT02002055.1	secreted	---NA---
AVOT02020385.1	secreted	Hypothetical protein *P. graminis* f. sp. *tritici*
AVOT02020447.1	secreted	---NA---
AVOT02002069.1	secreted	Hypothetical protein *P. graminis* f. sp. *tritici*
AVOT02002093.1	secreted	---NA---
AVOT02020745.1	secreted	Hypothetical protein *P. graminis* f. sp. *tritici*
AVOT02002131.1	secreted	Hypothetical protein *P. coronata* var. *avenae* f. sp. *avenae*
AVOT02000214.1	secreted	---NA---
AVOT02021158.1	nucleus	---NA---
AVOT02060111.1	secreted	Hypothetical protein *M. larici-populina*
AVOT02002154.1	secreted	NA
AVOT02021424.1	secreted	Hypothetical protein *P. sorghi*
AVOT02021436.1	secreted	---NA---
AVOT02021452.1	secreted	---NA---
AVOT02022200.1	secreted	---NA---
AVOT02002260.1	secreted	---NA---
AVOT02022493.1	secreted	---NA---
AVOT02002281.1	secreted	---NA---
AVOT02022744.1	secreted	---NA---
AVOT02022969.1	secreted	---NA---
AVOT02023170.1	secreted	---NA---
AVOT02023948.1	secreted	---NA---
AVOT02002490.1	secreted	Hypothetical protein *P. coronata* var. *avenae* f. sp. *avenae*
AVOT02024901.1	nucleus	---NA---
AVOT02034820.1	secreted	Hypothetical protein *P. coronata* var. *avenae* f. sp. *avenae*
AVOT02025268.1	secreted	---NA---
AVOT02025446.1	secreted	---NA---

AVOT02000262.1	secreted	---NA---
AVOT02002632.1	secreted	Sod_Cu domain-containing protein *P. graminis* f. sp. *tritici*
AVOT02002638.1	secreted	Hypothetical protein *P. triticina*
AVOT02026046.1	secreted	---NA---
AVOT02026132.1	cytoplasm	Chitin deacetylase *P. graminis* f. sp. *tritici*
AVOT02026248.1	secreted	---NA---
AVOT02026571.1	secreted	---NA---
AVOT02002709.1	secreted	---NA---
AVOT02002764.1	secreted	Hypothetical protein *P. graminis* f. sp. *tritici*
AVOT02027350.1	secreted	---NA---
AVOT02027480.1	secreted	---NA---
AVOT02027753.1	nucleus	Inhibitor I9 domain-containing protein
AVOT02002850.1	secreted	---NA---
AVOT02028049.1	secreted	Secreted protein *M. larici-populina*
AVOT02002870.1	secreted	---NA---
AVOT02028197.1	secreted	---NA---
AVOT02028690.1	secreted	Lipase_3 domain-containing protein *P. striiformis* f. sp. *tritici*
AVOT02002978.1	cytoplasm	---NA---
AVOT02029555.1	secreted	---NA---
AVOT02029676.1	secreted	---NA---
AVOT02029768.1	nucleus	Hypothetical protein *P. graminis* f. sp. *tritici*
AVOT02029819.1	secreted	---NA---
AVOT02029851.1	secreted	---NA---
AVOT02029865.1	cytoplasm	Sod_Cu domain-containing protein *P. graminis* f. sp. *tritici*
AVOT02000306.1	secreted	---NA---
AVOT02030500.1	endoplasmic reticulum membrane	Hypothetical protein *P. graminis* f. sp. *tritici*

AVOT02030686.1	secreted	---NA---
AVOT02030696.1	secreted	Dimer_Tnp_hAT domain-containing protein
AVOT02030755.1	secreted	---NA---
AVOT02030907.1	secreted	---NA---
AVOT02030930.1	secreted	Hypothetical protein *P. coronata* var. *avenae* f. sp. *avenae*
AVOT02030931.1	nucleus	Hypothetical protein *P. coronata* var. *avenae* f. sp. *avenae*
AVOT02031014.1	secreted	---NA---
AVOT02003245.1	secreted	---NA---
AVOT02032191.1	secreted	Hypothetical protein *P. graminis* f. sp. *tritici*
AVOT02032642.1	nucleus	---NA---
AVOT02032658.1	secreted	---NA---
AVOT02032858.1	secreted	Alpha-galactosidase *P. graminis* f. sp. *tritici*
AVOT02033137.1	secreted	---NA---
AVOT02033174.1	secreted	---NA---
AVOT02003396.1	secreted	Hypothetical protein *P. graminis* f. sp. *tritici*
AVOT02003413.1	secreted	---NA---
AVOT02033480.1	secreted	---NA---
AVOT02033602.1	secreted	---NA---
AVOT02034022.1	secreted	Hypothetical protein *P. triticina*
AVOT02034060.1	secreted	---NA---
AVOT02003479.1	secreted	---NA---
AVOT02034217.1	secreted	---NA---
AVOT02034331.1	secreted	---NA---
AVOT02034375.1	secreted	---NA---
AVOT02034526.1	secreted	---NA---
AVOT02034529.1	secreted	---NA---
AVOT02000354.1	secreted	---NA---

AVOT02003546.1	secreted	---NA---
AVOT02002550.1	cytoplasm	Hypothetical protein *P. coronata* var. *avenae* f. sp. *avenae*
AVOT02035464.1	secreted	Hypothetical protein *P. striiformis* f. sp. *tritici*
AVOT02035871.1	mitochondrion	Alpha/Beta hydrolase protein *Pseudomassariella vexata*
AVOT02036233.1	secreted	---NA---
AVOT02003738.1	secreted	---NA---
AVOT02003748.1	secreted	---NA---
AVOT02036836.1	chloroplast	---NA---
AVOT02037137.1	endoplasmic reticulum	Thioredoxin domain-containing protein *P. triticina*
AVOT02037844.1	secreted	---NA---
AVOT02037993.1	secreted	---NA---
AVOT02038520.1	cytoplasm	---NA---
AVOT02003988.1	plasma membrane	---NA---
AVOT02039004.1	secreted	Hypothetical protein *P. graminis* f. sp. *tritici*
AVOT02039196.1	secreted	Hypothetical protein *Aspergillus wentii*
AVOT02040016.1	secreted	---NA---
AVOT02040109.1	secreted	---NA---
AVOT02040127.1	secreted	---NA---
AVOT02040743.1	secreted	Hypothetical protein *P. sorghi*
AVOT02040813.1	secreted	---NA---
AVOT02022695.1	secreted	Hypothetical protein *M. larici-populina*
AVOT02041469.1	secreted	Secreted protein *M. larici-populina*
AVOT02004256.1	secreted	Hypothetical protein *P. coronata* var. *avenae* f. sp. *avenae*
AVOT02004259.1	cytoplasm	---NA---
AVOT02041605.1	secreted	---NA---
AVOT02004284.1	secreted	---NA---

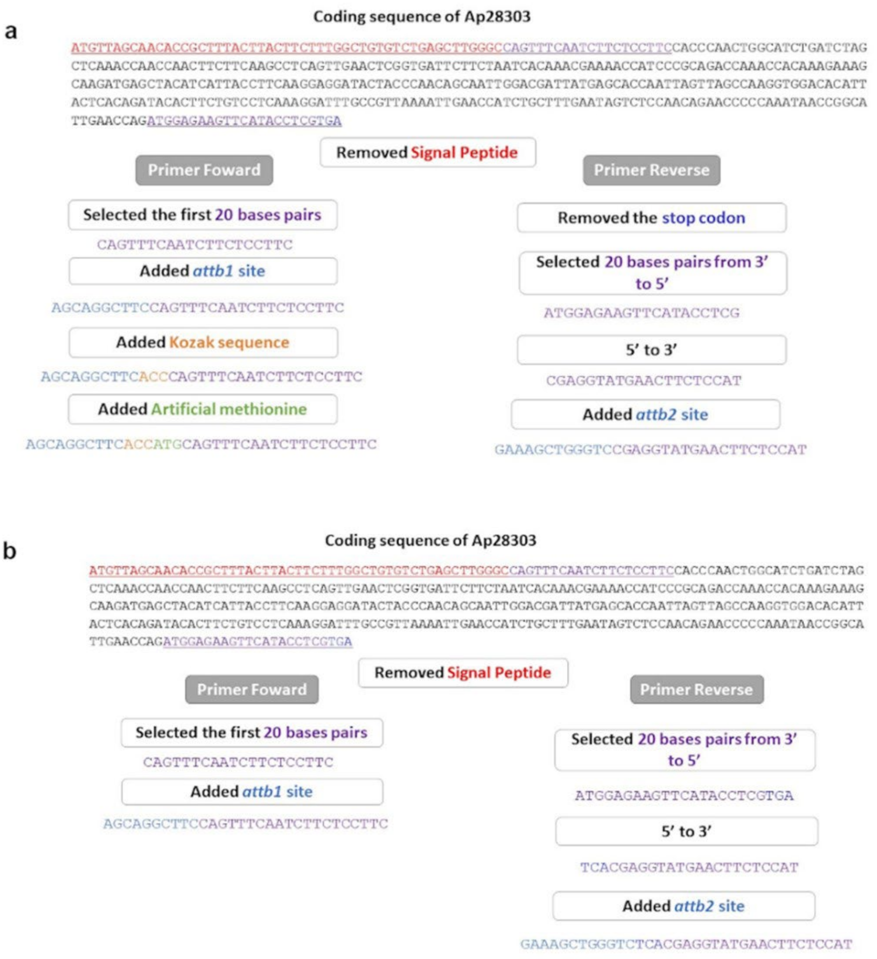

Supplementary Figure S1. Construction of primers for cloning experiments (a) construction of the primers to be recombined in the plasmid with the tag in C-terminal (b) construction of the primers to be recombined in the plasmid with the tag in N-terminal

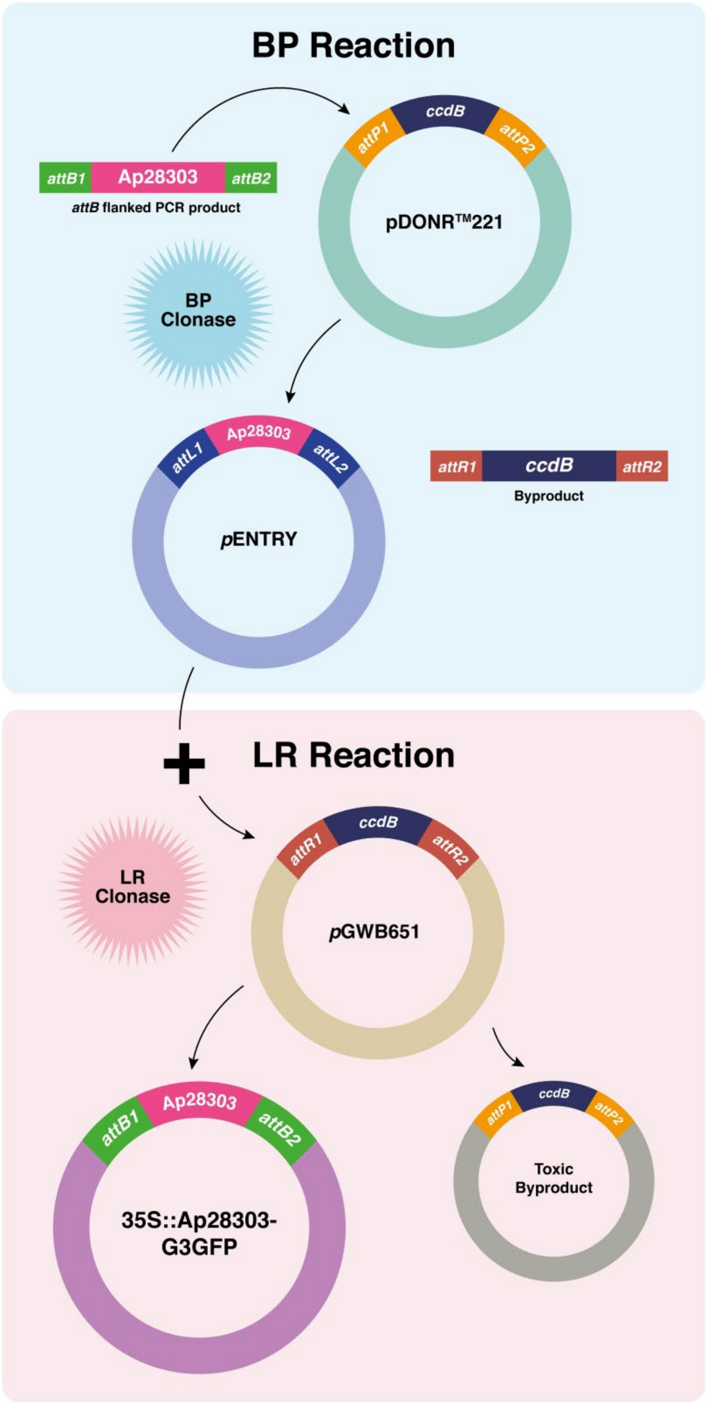

Supplementary Figure S2. Cloning procedures using Gateway System
